# Gross Hydrocele With Completely Buried Penis: A Case Report

**DOI:** 10.7759/cureus.47916

**Published:** 2023-10-29

**Authors:** Rahul D Pathrikar, Rohan R Kadak, Pranjali P Muley, Parikshit A Muley, Kamlesh J Wasnik, Nishikant Ingole

**Affiliations:** 1 Department of Urology, Fortis Hospital, Mumbai, IND; 2 Department of Surgery, Datta Meghe Medical College, Datta Meghe Institute of Higher Education and Research (DU), Wardha, IND; 3 Department of Physiology, Datta Meghe Medical College, Datta Meghe Institute of Higher Education and Research (DU), Wardha, IND; 4 Department of Pharmacology, Jawaharlal Nehru Medical College, Datta Meghe Institute of Higher Education and Research (DU), Wardha, IND

**Keywords:** testis, scrotal swelling, supra-pubic catheter, gross hydrocele, tunica vaginalis

## Abstract

A gross hydrocele is caused by fluid accumulation within a layer wrapped around the testicle, called the tunica vaginalis, derived from the peritoneum. A 65-year-old male complained of a non-tender, fluctuant bulge in his right scrotum despite having a fully buried penis, a large hydrocele, and urinary retention. After ultrasonography, the diagnosis was confirmed, and the patient underwent a successful surgical procedure that included a hydrocelectomy and tunica vaginalis excision. The patient reported few postoperative complications and a notable improvement in his quality of life. Surgery is a successful approach for treating gross hydrocele, with minimal morbidity and excellent cosmetic outcomes.

## Introduction

A pathological accumulation of serous fluid in the groin and pelvis caused by a variety of conditions, including illnesses or injuries, is known as gross hydrocele [1]. It is characterized by fluid accumulation in the tunica vaginalis, the sac that encloses the testis, which results in scrotal swelling [2]. Even though it is typically a benign condition, those affected may experience serious impairments in their daily lives and quality of life. For managing extensive hydrocele, surgical intervention involving hydrocelectomy and excision of the tunica vaginalis is a safe and successful treatment option with excellent cosmetic results and minor morbidity [3]. Despite the availability of the above-mentioned surgical treatment alternatives, many people avoid visiting a doctor because they are ashamed or afraid of having surgery. As a result, healthcare professionals may encounter difficulties in diagnosing and treating gross hydrocele. In this context, case studies help elucidate gross hydrocele's clinical manifestation, diagnosis, and treatment and emphasize the significance of early detection and fast treatment for the best results. In this report, we describe a case of severe hydrocele in a 65-year-old male with a fully buried penis for which supra-pubic catheterization was followed by surgery. The case emphasizes the gross hydrocele's clinical manifestation, radiological findings, surgical treatment, and influence on the patient's quality of life. This case report aims to raise awareness of gross hydrocele and stress the value of early diagnosis and fast treatment for the best results.

## Case presentation

A 65-year-old male visited the urology outpatient clinic complaining of a painless swelling in his scrotum that had persisted for the past two years. The patient did not give any history of any major trauma, such as recently having a fever or losing weight. The patient did not give any past history of medical or surgical intervention, and his overall general condition was good. The patient was not a smoker and denied having any significant family history.

Clinical findings: Physical examination revealed a significantly enlarged scrotum around 20 cm x 18 cm. The swelling was transilluminated, non-reducible, and painless. There was no sign of a hernia or varicocele, and the testicles could not be felt individually-the complete burial of the penis. The patient had urinary retention, and it was very difficult to put in the catheter. The patient had severe abdominal pain due to bladder distension, for which suprapubic catheterization was done.

Diagnostic assessment: An enormous fluid collection encircling both testicles was discovered during an ultrasound check of the scrotum. There was no sign of a solid tumor or inguinal hernia. The testicular dimensions on ultrasound were found to be 20 cm × 18 cm × 15 cm. Alpha-fetoprotein (AFP) and human chorionic gonadotropin (HCG), two serum tumor indicators, were within normal ranges.

**Figure 1:**
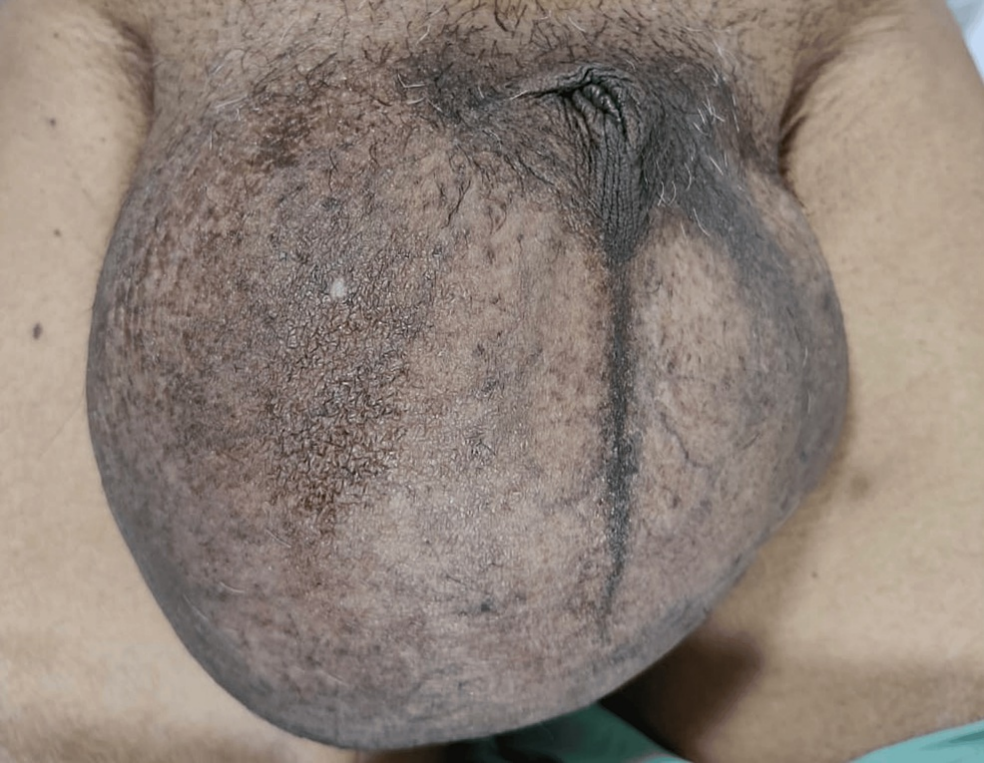
Gross hydrocele with a completely buried penis

Diagnosis: Gross hydrocele was identified based on the clinical findings, as depicted in Figure 1.

Therapeutic intervention: Surgery was recommended for the patient. Under spinal anesthesia, a midline scrotal incision was made to do the procedure. After draining the fluid, the tunica vaginalis was removed, and the fluid was sent for pathological investigations. The testicles were found to be consistent and of the appropriate size upon examination. A sterile dressing was applied to the scrotum, and absorbable sutures were used to close the wound.

Follow-up and outcome: The patient's postoperative course was uneventful, and he was discharged on the second postoperative day. The patient was instructed to refrain from any kind of strenuous exercise and wear scrotal support for four weeks. After six months of follow-up with the patient in the outpatient clinic, no swelling was observed.

## Discussion

Fluid accumulates around the testis in the tunica vaginalis due to the benign condition of gross hydrocele [4]. It is a problem that affects many adult men, with an incidence of up to 1%. Gross hydrocele can be pretty uncomfortable and distressing, even though it is typically painless, especially if the swelling progresses and interferes with daily activities [5]. A clinical examination is generally required to determine the presence of a non-tender, fluctuating mass in the scrotum, which is the hallmark of a large hydrocele. In addition to confirming the diagnosis, ultrasonography can rule out other disorders, such as epididymitis or testicular tumors [6]. When attempting to distinguish a large hydrocele from other scrotal lumps, a computed tomography (CT) scan or magnetic resonance imaging (MRI) may be required [7].

For patients with symptomatic or big hydroceles, surgery is the recommended course of treatment for gross hydroceles. The hydrocelectomy, or removal of the hydrocele sac and tunica vaginalis, is a standard surgical procedure [8]. This operation has a low risk of complications and positive cosmetic results; it cannot be done under local anesthesia but under spinal or general anesthesia [9].

The benefits of surgical care for gross hydrocele include symptom reduction, avoidance of complications such as testicular torsion, and enhancement of quality of life [10]. However, surgery has certain possible dangers, including infection, bleeding, and injury to nearby tissues. Potential advantages and restrictions of surgery, along with other hazards, should be discussed with patients. Surgical intervention comprising hydrocelectomy and excision of the tunica vaginalis is a safe and effective therapy option for managing severe hydrocele, with minimal morbidity and great aesthetic results.

## Conclusions

Early diagnosis and treatment are essential for preventing complications and improving the patient outcomes. Surgical intervention comprising hydrocelectomy and excision of the tunica vaginalis is a safe and effective therapy option for controlling severe hydrocele. With the appropriate treatment, people with gross hydrocele can enhance their quality of life and avoid negative outcomes. 
